# Evaluation of serological assays for *intra vitam* diagnosis of bovine tuberculosis in water buffalo (*Bubalus bubalis*)

**DOI:** 10.3389/fmicb.2025.1684425

**Published:** 2025-11-12

**Authors:** Sara Giovannozzi, Alessandra Martucciello, Mercedes Domínguez Rodríguez, Inmaculada Moreno Iruela, Mery Boifava, Lorena Schiavo, Anna Viscito, Giovanni Parisio, Nicoletta Vitale, Javier Bezos, Esterina De Carlo, Maria Beatrice Boniotti

**Affiliations:** 1 National Reference Centre for Bovine Tuberculosis (CNR-bTB), Istituto Zooprofilattico Sperimentale della Lombardia e dell’Emilia-Romagna (IZSLER), Brescia, Italy; 2 Istituto Zooprofilattico Sperimentale del Mezzogiorno, National Reference Centre for Hygiene and Technologies of Water Buffalo Farming and Productions, Salerno, Italy; 3 Unidad de Inmunología Microbiana, Centro Nacional de Microbiología, Instituto de Salud Carlos III, Madrid, Spain; 4 Istituto Zooprofilattico Sperimentale del Piemonte, Liguria e Valle d'Aosta, Torino, Italy; 5 Animal Health Department of Veterinary Faculty and VISAVET Health Surveillance Centre, Complutense University of Madrid, Madrid, Spain

**Keywords:** *Mycobacterium bovis*, tuberculosis, ELISA, PPDB, P22, diagnosis, water buffalo

## Abstract

**Introduction:**

Bovine tuberculosis (TB) in water buffalo (*Bubalus bubalis*) is primarily diagnosed using *intra vitam* tests, as the intradermal tuberculin test (IDT) and the interferon-gamma release assay (IGRA), both of which detect cell-mediated immunity (CMI). However, a subset of infected animals fails to mount a detectable CMI response, posing a significant risk of undetected transmission. Serological tests assessing the humoral immune response could provide a valuable complementary tool for identifying infected animals that escape detection through traditional CMI-based assays.

**Methods:**

This study analyzed 895 serum samples from water buffaloes, including 393 from TB-free herds and 502 from TB-infected herds. Animals from TB-free herds were tested using IDT and an ELISA assay, whereas those from infected herds were also tested using IGRA. We developed an ELISA assay targeting MPB70, MPB83, ESAT6, CFP10, PPDB, and P22 antigens to investigate the role of the humoral response in TB diagnosis.

**Results and discussion:**

The ELISA showed a specificity of 98.2%. However, sensitivity differed based on the antigen used: among the most reactive proteins, sensitivity was 67.5% for MPB70, 69.8% for P22, and 74.4% for PPDB. Moreover, approximately 70% of samples with discordant IDT and IGRA results, as well as those with positive IDT but inconclusive IGRA results, tested positive by serology, highlighting the potential of antibody-based detection to improve TB diagnosis in buffaloes. Our findings suggest that integrating serological testing with standard diagnostic methods could enhance the detection of infected animals, ultimately contributing to better TB control in buffalo populations.

## 1 Introduction

The *Mycobacterium tuberculosis* complex (MTBC), mainly *Mycobacterium bovis* and *Mycobacterium caprae*, is responsible for causing tuberculosis (TB) in cattle, buffaloes, other wild and domestic animal species, as well as in humans (Grange, 2001; De Lisle et al., 2001). Transmission can occur through direct contact with infected animals or indirectly via contaminated equipment, water, or food (Kaneene and Thoen, 2004). Buffaloes, in particular, are highly social animals that are typically raised in intensive systems where they are free to roam, though not kept on pasture. They are also better adapted than cattle to cope with heat stress, often spending extended periods wallowing in mud to regulate their body temperature. This behavior may facilitate the spread of *M. bovis* within the herd (Carneiro et al., 2019).

TB poses a significant threat to both public health and the economy due to its presence in multiple animal species and the risk it poses to animal products intended for human consumption. The economic impact of bovine tuberculosis (bTB) in water buffalo (*Bubalus bubalis*) is substantial, especially in regions where buffalo farming is vital to local livelihoods. For instance, in Southern Italy, particularly the Campania region, water buffalo herds are essential for the production of buffalo mozzarella (Martucciello et al., 2020; Martucciello et al., 2024) and are considered animals of high commercial and genetic value. TB in cattle leads to reduced productivity, including lower milk yields and slower weight gain, and requires the culling of infected animals, thereby intensifying the economic burden (Ramos et al., 2015). In Campania alone, more than €20 million was spent annually on farmer compensation for culled animals, underlining the substantial financial toll of the disease. These losses include reduced genetic diversity due to slaughtered stock and the mandatory heat treatment of milk from infected herds, which adversely affects product value and trade potential (Martucciello et al., 2020; Martucciello et al., 2024).

For these reasons, strict eradication programmes are enforced both at EU and national level, based on surveillance, culling of infected animals, and movement restrictions (European Union, 2024; Gazzetta Ufficiale, 2024).

The diagnosis of TB in buffaloes represents a crucial element in disease control and eradication programs. *Intra vitam* testing allows infected animals to be identified before they show clinical signs, preventing the spread of *M. bovis* within the herd and limiting risks to public health.

Over the past century, the main antigens used for TB diagnosis have been complex whole-cell lysates and poorly defined culture filtrates, particularly tuberculin purified protein derivatives (PPDs) obtained from heat-killed mycobacterial cultures. These complex preparations provide a wide variety of antigens that can be presented to lymphocytes, reflecting the range of antigens to which the host is exposed during infection. Although PPD ensures excellent sensitivity (Se) in diagnostic tests, it shows limited specificity (Sp) due to shared epitopes with other mycobacteria and closely related bacteria (Palmer and Waters, 2006). Available *intra vitam* diagnostic tests in buffalo farms are the intradermal (IDT) tests and the interferon-gamma release assay (IGRA), which are based on PPDs. The IDT can be of two types: the single intradermal tuberculin test (SITT) with *M. bovis* PPD (PPDB), and the comparative intradermal tuberculin test (CITT) by using a second injection with *M. avium* PPD (PPDA). Differences in immune responses between water buffaloes and cattle to *M. bovis* are probably influenced by species-specific factors. Water buffaloes exhibit immune traits characterized by reduced Se and Sp in diagnostic tests such as the IDT, which may yield false negatives due to malnutrition or false positives due to exposure to non-tuberculous mycobacteria (Martucciello et al., 2020). Carrying out SITT and CITT tests in water buffalo involves technical difficulties represented by the black color and the natural thickness of the buffalo skin into which the tuberculin is inoculated. These tests are based on a delayed-type hypersensitivity reaction, where tuberculin is injected intradermally and the immune response is evaluated by measuring skin thickening at the injection site after 72 h. The reliability of the test is strictly linked to manual skill and precision of tuberculin injections. Moreover, skin thickening reading, even if it is carried out using a caliper, is still subjective. In the IGRA test, PPDA and PPDB are used to stimulate T lymphocytes from peripheral blood. The release of gamma-interferon during the incubation period is measured. It is therefore important to preserve cell viability and ensure a sufficient interval from the IDT, which could otherwise interfere with the immune system (Martucciello et al., 2020). This aligns with the official IGRA protocol adopted in the European Union: the European Standard Operating Procedures (SOP) of the EURL-TB [SOP/004/EURL (VISAVET, 2021)]. These *intra vitam* tests therefore investigate the cell-mediated immunity (CMI) response to *M. bovis*, however, some infected animals may be anergic and fail to respond to IDT and/or IGRA (Fontana et al., 2018; McCallan et al., 2017; Pollock et al., 2005). Chronic form of TB infection often results in a suppressed CMI response, which has renewed interest in detecting such immunosuppressed animals by revealing specific antibodies against *M. bovis* (Alvarez, 2021). Consequently, the possibility that humoral immunity could support the accurate identification of the infectious status in animals has been explored (Carneiro et al., 2021; Griffa et al., 2020). Serological tests offer several advantages over CMI-based tests such as IDT and IGRA. First, they require only a single sampling, allowing each animal to be captured once. Second, they provide results within a few hours. Third, they enable the simultaneous analysis of many samples, lowering per-sample consumable costs. Nevertheless, their Se is often low, particularly in recently infected animals (Bezos et al., 2014; Fontana et al., 2018). The ability to detect *M. bovis* by serology indeed depends on the stage of infection, if the animal has developed a detectable humoral response, as well as on the prevalence of the disease (van der Heijden et al., 2020). Joseph B. et al. supported the hypothesis that high of antibody levels were associated with mycobacterial shedding and correlated with the infectious stage of TB, and proposed that combining ELISA with PCR could be useful for identifying buffaloes unresponsive to SITT (Joseph et al., 2021). Another study evaluated the serological response to MPB70 in bovine and water buffalo, exploiting an immunologic assay for detecting anergic animals (Zarden et al., 2013). Borham et al. (2022) applied a commercial ELISA kit to a mixed group of cows and buffaloes, observing no significant difference in TB prevalence between species. Their results showed high Se but low Sp; however, the assay was able to detect 80% (4/5) of animals with TB lesions at the slaughterhouse.

The development of serological assays to detect antibodies against *M. bovis* infection began with the first ELISA based on PPDB, and later expanded to include proteins such as MPB70 and MPB83, which proved to be specific for *M. bovis* (Ritacco et al., 1987; Harboe et al., 1990; Klepp et al., 2024). These two proteins are major components of PPDB, and immune reactivity to them may increase following IDT testing (Lyashchenko et al., 2004). ESAT6 and CFP10 are potent T-cell–stimulating antigens secreted by *M. tuberculosis* and *M. bovis* (Klepp et al., 2024; Vordermeier et al., 2011; Encinas et al., 2018), and are primarily used in IGRA assays (van Pinxteren et al., 2000; Buddle et al., 1999; Aagaard et al., 2006). These proteins are considered key virulence factors (Belogorodtsev et al., 2021). P22, in contrast, is a protein complex obtained by purifying PPDB through affinity chromatography (Infantes-Lorenzo et al., 2017). MPB70, MPB83, CFP10, ESAT6, and PPDB have already been tested for TB serodiagnosis in cattle (Fontana et al., 2018; Moens et al., 2023), showing that ELISA can offer various result-interpretation criteria, with Se and Sp comparable to other serological tests (Bezos et al., 2014).

The potential contribution of ELISA to the eradication of bTB is promising. While numerous studies have been conducted in recent years on different animal species, data on buffaloes remain scarce, and species-specific test validation is needed.

The present study aims to evaluate MPB70, MPB83, ESAT6, CFP10, PPDB, and P22 as potential antigens for an ELISA test on water buffalo samples. The results could support the implementation of improved diagnostic strategies, contributing to more effective TB control measures and outbreak management.

## 2 Materials and methods

### 2.1 Design of the study

A total of 895 buffaloes were included in this study: 393 animals were from four officially tuberculosis-free (OTF) herds, certified as TB-free for at least 6 years, and 502 were from 78 herds involved in TB confirmed outbreaks (Table 1). The animals were sampled between 2017 and 2019. For the purposes of this study, an outbreak was considered confirmed when at least one animal in the herd tested positive using the IDT and/or the IGRA, with subsequent confirmation by *M. bovis* isolation. The IDT was applied as described by Martucciello et al. (2024) and was considered positive when the increase in skinfold thickness at the bovine PPD injection site was ≥4 mm. The IGRA was initially performed using two pairs of bovine and avian PPDs (Italian and Lelystad) and interpreted according to criteria 3 described by Martucciello et al. (2020), which defines a sample as positive when the net PPDB/PPDA ratio is ≥1.1 in optical density (OD) and both PPDB and PPDA are at least twice those of the PBS control; samples with a ratio between 0.9 and 1.1 OD were considered inconclusive. In those cases, criterion 2 was applied, using only Lelystad PPDs and considering a sample positive when the PPDB–PPDA difference was ≥0.05 OD and PPDB was ≥2 × PBS.

**Table 1:** Summary of diagnostic test outcomes in 895 animals selected from TB-free and TB-infected herds included in the study.

TB status	Samples	Lesions	Culture isolation^+^	IGRA^+^	IDT^+^
Tb free	393	0	0	0	0
Tb infected	502	373	62	462	441
Post-mortem +*	344^a^	320	62	340	342
Post-mortem − **	105	0	0	88	82

*(IDT^+^, IGRA^+^, lesion or culture isolation^+^), **(IDT^+^ or IGRA^+^, lesion and culture isolation^−^).

a Group of samples used for assessing Se.

Buffaloes from TB-free herds were tested for IDT and indirect ELISA. Buffaloes from TB outbreaks were tested for IDT, IGRA, and indirect ELISA. In cases where one or more animals tested inconclusive and/or positive to the SITT, at least 42 days after PPDB inoculation they were subjected to CITT and IGRA testing. Additionally, blood samples for ELISA testing were collected at least 42 days after the PPDB inoculation of the SITT (typically between 50 and 237 days), and 72 h after the PPDB inoculation of the CITT. Slaughtered animals were checked *post-mortem* by the competent veterinary service to search for tuberculin-like lesions, and samples were collected for microbial isolation. IDT and IGRA tests were performed as described before (Martucciello et al., 2020). The culture isolation of MTBC was performed as described by Ferrari et al. (2024).

The ELISA diagnostic accuracy was determined by using a subset of the 895 buffaloes initially enrolled in the study (393 from OTF herds and 502 from herds with confirmed bTB outbreaks). This subset comprised a total of 737 animals, including 344 buffaloes from infected herds who met the strict criteria for being classified as truly positive. These criteria included: (i) a positive result from microbial isolation, considered the gold standard for TB diagnosis (Albernaz et al., 2015; Alvarez, 2021), or (ii) concurrent positivity to both the IDT and IGRA tests along with the presence of TB-like lesions at post-mortem inspection. Animals from infected herds that did not meet either of these criteria were excluded from the analysis. The diagnostic accuracy analysis included all 393 buffaloes from officially TB-free (OTF) herds, classified as truly negative, to estimate test Sp; these animals also underwent IGRA testing to confirm their TB-negative status.

Finally, in the IGRA test, negative samples showing an OD difference between Pokeweed mitogen (PWM) and PBS of < 0.5 were considered invalid.

### 2.2 Indirect ELISA

A multi-antigen ELISA test, based on the purified recombinant proteins MPB70, MPB83, ESAT6, CFP10 and the PPDB, was performed as described by Fontana et al. (2018), while P22 was produced by the Instituto de Salud Carlos III, Madrid, Spain, as described before (Infantes-Lorenzo et al., 2017). The coating of the microplates (Maxisorp, Nunc, Rochester, NY, USA) was with 1 μg/mL of MPB70, MPB83, ESAT6 and CFP10, 5 μg/mL of PPDB, and 10 μg/mL of P22 in carbonate buffer at pH 9.6 and with an overnight incubation at 4 °C. The plates were washed with buffer phosphate-buffered saline (PBS) 1 × (30 mM phosphate buffer pH 7.2, 150 mM NaCl and 2 m M EDTA) with 0.05% Tween-20 and blocked with 2% skimmed powdered milk in PBS-T buffer [1X PBS (30 mM phosphate buffer pH 7.2, 150 mM NaCl and 2 mM EDTA) and 0.1% Tween-20]. After three washes, the plates were blocked with 50 μL for MPB70, MPB83, ESAT6, CFP10, and PPDB, whereas 100 μL for P22 of 2% skimmed powdered milk (Marvel Original Dried Skimmed Milk, Freepost Premier Foods, Ireland) in PBS-T buffer for 1 h at room temperature with agitation. After washing the plates three times, 50 μL of serum was diluted 1/50 in PBS-T buffer with 1% yeast extract (Oxoid, Altrincham, Cheshire, England), and added to two coated wells and one uncoated. For P22, sera were diluted 1/100 in PBS-T solution with 2% of skimmed powdered milk. After incubation for 1 h at 37 °C and three washes, 50 μL of a 1/10,000 dilution of horseradish peroxidase-conjugated antibody [mAb 1G10; (Brocchi et al., 2006)] in PBS-T buffer with 1% yeast extract was added for MPB70, MPB83, ESAT6, CFP10, and PPDB; the antibody was diluted just in PBS-T buffer for P22. The murine mAb 1G10 recognizes bovine IgG1, and cross-reacts with IgG of ruminant species, including water buffalo (Brocchi et al., 2006). After 1 h at 37 °C and three washes, 0.05 mg/mL of o-phenylenediamine (Sigma, Milan, Italy) and 0.02% v/v of H_2_O_2_ were added, and the reaction was stopped with 50 μL of 2 N H_2_SO_4_ (Merck, Darmstadt, Germany) after 5 min. OD was measured at a wavelength of 492 nm by a Multiskan Ascent spectrophotometer (MTX, USA). In each plate, positive and negative controls were used to check the ELISA reaction. ELISA results were expressed as positive percentage (PP%) using the formula: (average OD sample—OD blank/average OD positive control—OD blank) × 100. Cut-off values were calculated using Receiver Operating Characteristic (ROC) curve analysis, as described in the Statistical Analysis section, by setting the Sp to be at least 98.2%. The term “multi-antigen ELISA” refers to six independent indirect ELISAs, each performed in separate wells coated with a single antigen (MPB70, MPB83, ESAT6, CFP10, PPDB, or P22). Each assay was interpreted individually based on its own cut-off value, and no combined or parallel interpretation of results was applied.

### 2.3 Statistical analysis

The diagnostic accuracy of the ELISA tests was then assessed on a subset of 737 buffaloes, which were classified as true positives and true negatives based on the aforementioned criteria. The estimation of Se was conducted on buffaloes classified as true positive (*n* = 344), whereas the estimation of Sp was conducted on all buffaloes from OTF herds (*n* = 393).

Results expressed as PP% (sample/positive control × 100) were treated as continuous variables and analyzed using receiver operating characteristic (ROC) curves to determine optimal cut-off values. For each test, the following performance indices were calculated: Se, Sp, proportion of false positives, proportion of false negatives, accuracy, Youden index and the area under the ROC curve (AUC).

The agreement between the six antigens (MPB70, MPB83, ESAT6, CFP10, PPDB, and P22) and the reference classification (negative for animals from TB-free herds and positive for animals with culture confirmation or with both IDT and IGRA positivity and tuberculous-like lesions) was evaluated in 737 animals using Cohen’s Kappa coefficient and McNemar’s test, implemented via the DescTools package in R (Signorell, 2014). A Kappa value of 1 indicates perfect agreement, while a value of 0 indicates no agreement beyond chance. To assess whether a statistically significant difference existed between the number of false positives and false negatives, thus identifying potential asymmetry in misclassification between test results and the reference classification, the McNemar test was applied.

In TB-positive herds, the multi-antigen ELISA was evaluated by comparing its results with those of *intra vitam* (IV) diagnostic tests (IDT and IGRA) and post-mortem (PM) findings. To further assess the diagnostic performance of the ELISA, the proportion of animals testing positive to the antigens MPB70, ESAT6, PPDB, and P22 was compared between the IV+PM+ subgroup (positive to both *intra vitam* and *post mortem* tests) and the IV+PM− (positive to *intra vitam* but negative to *post mortem* tests) subgroup using Fisher’s exact test. The same analysis was applied to the IDT^−^/IGRA^+^ and IDT^+^/IGRA^−^ groups, in order to evaluate the ability of the ELISA to detect infection in animals with discordant cell-mediated immune responses.

Optimal cut-off values were identified with the Youden index (*J* = Se + Sp − 1), which determines the threshold that best balances Se and Sp. Each point on the ROC curve represents a potential PP% cut-off along with its corresponding Se, Sp, and Youden’s index. To guide the selection of the optimal values, a Sp threshold was fixed at the first value above 98%, which was considered an acceptable level. The AUC was used to assess overall test performance and discriminatory ability. Confidence intervals (95%) were calculated for sensitivity, specificity, and AUC using exact binomial methods.

The repeatability of ELISA was made by selecting three levels of antibody concentration (high, medium and low/negative) defined by their PP%. Intra-assay repeatability was evaluated with three independent reactions on the same day for each antigen. Inter-assay repeatability was assessed by repeating the test 20 times over several days for each antigen, covering all three levels of antibody concentration. Repeatability intra and inter-assay were calculated using the coefficient of variation (CV%) of PP% corresponding to (standard deviation/mean) × 100.

All analyses were conducted using R software (version 4.4.0; R Core Team, 2024) with the “pROC” package (Robin et al., 2011).

## 3 Results

### 3.1 Diagnostic performance of the TB-ELISA

To assess the accuracy of the TB-ELISA, a total of 737 buffalo serum samples were included in the ROC curve analysis (Table 1), the results of which are reported in Table 2. Among these, 393 samples from TB-free herds were classified as negative, and 344 samples from TB-infected herds were classified as positive.

**Table 2:** Indices of test accuracy for the six ELISA antigens based on optimal cut-off values.

Test	%FP	%FN	SE (CI 95%)	SP (CI 95%)	Accuracy	AUC	Cut-off	Y
MPB70	19.3%	8.4%	91.6% (82.3–94.2%)	80.7% (66.9–84.5%)	85.8%	0.93 (0.91–0.95)	1.17	72.3%
MPB83	21.9%	21.5%	78.5% (69.2–82.6%)	78.1% (58.3–82.2%)	78.3%	0.82 (0.79–0.85)	1.01	56.6%
ESAT6	15.0%	37.2%	62.8% (55.2–68%)	85% (77.6–89.6%)	74.6%	0.73 (0.69–0.77)	0.4	47.8%
CFP10	15.3%	63.4%	36.6% (25.3–41%)	84.7% (71.8–88.3%)	62.2%	0.55 (0.51–0.6)	1.01	21.3%
PPDB	12.5%	7.6%	92.4% (84.6–95.1%)	87.5% (76.8–90.6%)	89.8%	0.95 (0.93–0.97)	1.17	79.9%
P22	12.2%	14.5%	85.5% (79.4–89.5%)	87.8% (81.9–91.6%)	86.7%	0.93 (0.91–0.95)	7	73.3%

The table shows the percentage of false positives (%FP) and false negatives (%FN), sensitivity and specificity with their 95% confidence intervals (Se and Sp, 95% CI), overall accuracy, area under the ROC curve (AUC, 95% CI), selected cut-off values (PP%), and Youden’s index (Y). ROC curve results are presented together with Youden’s index to identify the cut-off that provides the best compromise between sensitivity and specificity.

Se, Sp, and optimal cut-off values for each antigen were initially determined through ROC curve analysis and the Youden index. At these optimal thresholds, PPDB showed the highest diagnostic performance, with a Se of 92.4% and Sp of 87.5%, followed by MPB70 (91.6% Se, 80.7% Sp) and P22 (85.5% Se, 87.8% Sp). These results reflect a good trade-off between Se and Sp, as shown in Table 2. However, since some antigens showed relatively low Sp at the optimal cut-offs, which could compromise their utility in field diagnosis, Sp was fixed at 98.2% for all tests (Table 3) to ensure a high capacity for identifying true negatives. Under this constraint, PPDB maintained the highest Se (63.4, 95% CI: 51.7%–74.4%), followed by P22 (61.3, 95% CI: 48.8%–69.8%) and MPB70 (58.7, 95% CI: 52.9%–67.5%). These findings confirm PPDB and P22 as the most reactive and diagnostically reliable antigens under high-specificity conditions. To increase Se, multiple combinations of antigens were analyzed (Supplementary Table S1). A higher Se could be obtained by combining either P22 or PPDB with MPB70 (0.68 or 0.67), or by combining P22 with CFP10 and ESAT6 (0.66), although in this latter case the Sp was slightly lower (0.94). The four recombinant proteins (MPB70, MPB83, ESAT 6 and CFP10) together provided a Se that is comparable to that of PPDB alone (0.64 than 0.63).

**Table 3:** Indices of test accuracy for the six ELISA antigens with specificity fixed at 98.2%.

Test	%FP	%FN	SE (CI 95%)	SP (CI 95%)	Accuracy	Cut-off	Y
MPB70	1.8%	41.3%	58.7% (52.9–67.5%)	98.2% (96.4–99.3%)	79.6%	9.77	56.9%
MPB83	1.8%	58.4%	41.6% (29.9–48.3%)	98.2% (96.4–99.3%)	71.8%	12.32	39.8%
ESAT6	1.8%	67.2%	32.8% (27.6–40.7%)	98.2% (96.4–99.3%)	67.7%	5.94	31.0%
CFP10	1.8%	86.0%	14% (10.2–18.6%)	98.2% (96.4–99.3%)	58.9%	6.91	12.2%
PPDB	1.8%	36.6%	63.4% (51.7–74.4%)	98.2% (96.4–99.3%)	82.0%	13.77	61.6%
P22	1.8%	38.7%	61.3% (48.8–69.8%)	98.2% (96.4–99.3%)	80.9%	24.83	59.5%

The table shows the percentage of false positives (%FP) and false negatives (%FN), sensitivity (Se) and specificity (Sp) with their 95% confidence intervals (95% CI), overall accuracy, area under the ROC curve (AUC, 95% CI), selected cut-off values (PP%), and Youden’s index (Y).

Agreement between the ELISA results and the reference classification (true positives and true negatives) was evaluated using Cohen’s Kappa and McNemar’s test (Supplementary Table S2). The highest agreement was observed for PPDB (Kappa = 0.796), followed by P22 (Kappa = 0.733) and MPB70 (Kappa = 0.719), indicating substantial concordance with the reference standard. Moderate agreement was observed for MPB83 and ESAT6, while CFP10 showed poor agreement (Kappa = 0.22), consistent with its low sensitivity.

McNemar’s test revealed a statistically significant asymmetry in misclassification for most antigens (*p* < 0.05), except for MPB83 (*p* = 0.3845) and P22 (*p* = 0.9195) (Supplementary Table S2). This suggests that P22 produced a balanced rate of false positives and false negatives, further supporting its diagnostic robustness.

Among the 344 positive sera analyzed, reactivity to one or more antigens varied considerably (Supplementary Table S3). The majority of samples (*n* = 253) reacted to a single antigen, while a substantial number showed broader responses: 217 samples reacted to two antigens, 192 to three, 145 to four, 94 to five, and 34 sera were reactive to all six antigens tested. To further explore antigen co-reactivity, we analyzed the frequency of positive responses to pairs of antigens among TB-positive sera (Supplementary Table S4). The most frequent co-reactivity was observed for the PPDB/P22 combination, which was positive in 191/344 TB-positive sera (55.5%). Similarly, MPB70 in combination with either PPDB or P22 showed high reactivity, with 191 (55.5%) and 180 (52.3%) sera testing positive, respectively.

The repeatability of the TB-ELISA was determined by evaluating intra- and inter-assay variation through multiple replicates, as described in the Materials and Methods section. Variability was expressed as the coefficient of variation (CV%). Intra-assay CVs indicated good repeatability, particularly at high and medium antibody levels, with values ranging from 3.08% to 7.56% for MPB70, 1.18% to 1.65% for MPB83, 1.54% to 1.58% for ESAT6, 2.34% to 4.55% for CFP10, 0.42% to 1.34% for PPDB, and 1.91% to 7.67% for P22 (Table 4). As expected, higher variability was observed at lower antibody levels. Inter-assay variability, assessed across different days, showed acceptable repeatability, though with generally higher CVs compared to intra-assay results (Table 4). Ranges were 7.17%–13.81% for MPB70, 3.03%–8.41% for MPB83, 4.42%–9.50% for ESAT6, 11.42%–14.70% for CFP10, 3.19%–10.82% for PPDB, and 5.97%–10.29% for P22. Higher variability was observed at lower antibody levels, in particular for MPB70 (21.95%). All values fell within commonly accepted thresholds for ELISA assays, with CVs below 15%–20% generally considered acceptable for high and medium optical density, and below 25% for low levels.

**Table 4:** Intra-assay and inter-assay repeatability of high, medium, and low antibody levels (CV%).

Repeatability	Antigen	CV%		
		High	Medium	Low
Intra	MPB70	7.17	13.81	21.95
	MPB83	3.03	8.41	14.13
	ESAT6	4.42	9.50	11.91
	CFP10	14.70	11.42	13.95
	PPDB	3.19	10.82	10.03
	P22	10.29	5.97	14.03
Inter	MPB70	3.08	7.56	23.33
	MPB83	1.65	1.18	0.83
	ESAT6	1.54	1.58	2.25
	CFP10	2.34	4.55	9.10
	PPDB	1.34	0.42	0.84
	P22	7.67	1.91	9.08

### 3.2 Diagnostic value of TB-ELISA in positive herds

The multi-antigen ELISA was evaluated in infected herds, and the results were compared with those obtained from *intra vitam* (IDT and IGRA) and *post mortem* diagnostic tests.

Among animals with positive *intra vitam* tests (*n* = 501), serological analysis revealed notable differences depending on *post mortem* confirmation (Table 5). Interestingly, even in the absence of *post mortem* confirmation, a substantial proportion of animals (IV+/ PM−) showed seroreactivity to at least one antigen (41% to MPB70, 38% to MPB83, 43% to PPDB, and 30% to P22), indicating that serology can support and reinforce *intra vitam* test results. Notably, the fact that most of these samples tested positive for multiple antigens strongly suggests that these are true positives rather than false-positive results. In animals with both *intra vitam* and *post mortem* positivity, higher reactivity rates were observed across all antigens. In particular, antigens MPB70, ESAT6, PPDB, and P22 show a significantly higher positivity rate in the IV+PM+ group than in IV+PM− and the difference resulted statistically significant (exact test of Fisher *p < 0.01*). Conversely, MPB83 and CFP10 do not show statistically significant differences between the two groups (*p > 0.05*). All McNemar *p*-values are < 0.001, indicating asymmetric discordance: discrepancies between antigen and IV/ PM results are not random but directional. Kappa values are very low across all antigens (maximum 0.214), indicating poor agreement beyond chance. Raw agreement is also limited, especially for CFP10 (14%) and ESAT6 (28.8%). The low Kappa values and limited raw agreement indicate that antigen-based tests and IV/PM classifications assess different aspects of infection status and are not fully interchangeable. However, the directional nature of discordances (as shown by McNemar’s test) and the significantly higher reactivity of specific antigens in confirmed cases suggest that the antigenic tests provide complementary information. P22 in combination with PPDB or MPB70 showed a raw agreement >63% and a Kappa value between 0.18 and 0.19. The Kappa values remain low (<0.20), indicating that the agreement regarding the IV+/PM– and IV+/PM + combinations is negligible. The combinations of three of four antigen decrease the agreement level (Table 5).

**Table 5:** ELISA results in TB positive herds: agreement between *intra vitam* and *post mortem* tests.

Antigens	IV+/PM−	IV+/PM+	IV+/PM−%	IV+/PM+ %	Fisher_p	McNemar_p	Kappa	Raw agreement
MPB70	43	235	41.0%	59.3%	0.0009	0.0000	0.13	59.3%
MPB83	40	160	38.1%	40.4%	0.7	0.0000	0.01	44.9%
ESAT6	18	124	17.1%	31.3%	0.005	0.0000	0.08	42.1%
CFP10	11	58	10.5%	14.6%	0.3	0.0000	0.02	30.3%
PPDB	45	248	42.9%	62.6%	0.0003	0.0000	0.145	61.5%
P22	31	240	29.5%	60.6%	0.0000	0.0000	0.2	62.7%
MPB70/P22	46	271	43.8%	68.4%	0.0000	0.0000	0.19	65.9%
PPDB/P22	48	272	45.7%	68.7%	0.0000	0.0000	0.181	65.7%
ESAT6/CFP10/P22	42	257	40.0%	64.9%	0.0000	0.0000	0.186	63.9%
MPB70/MPB83/ESAT6/CPF10	58	256	55.2%	64.6%	0.09	0.0000	0.07	60.0%

IV, *intra vitam*; PM, post mortem.

Among sera collected from animals with discordant *intra vitam* (IDT/IGRA) and *post mortem* results, differential reactivity to individual antigens was observed, reflecting heterogeneity in immune response profiles depending on the diagnostic category.

**Figure 1:**
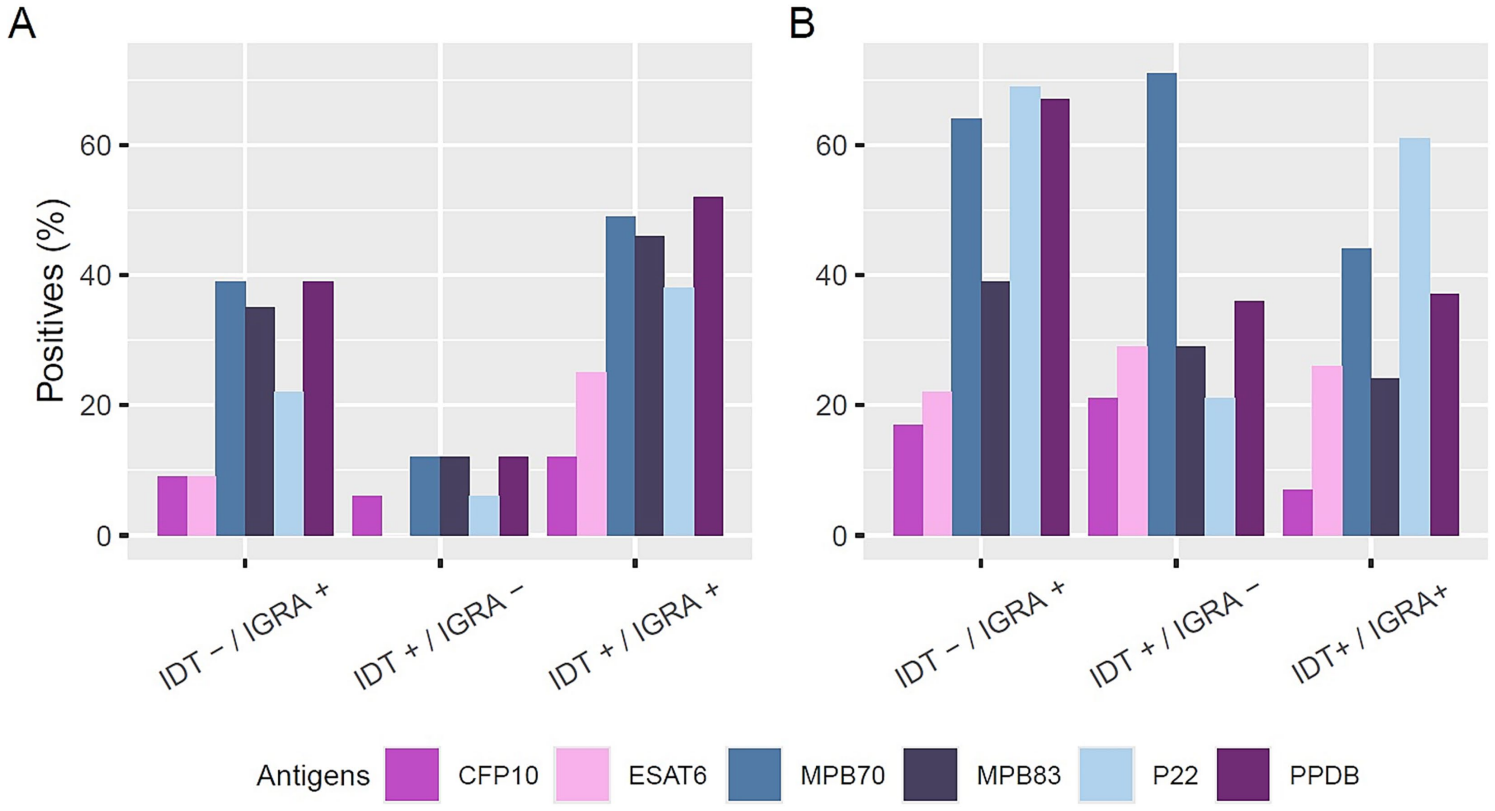
Antibody reactivity to different antigens in positive sera without **(A)** and with **(B)** post-mortem confirmation of infection. Sera are grouped based on intradermal test (IDT) and interferon-gamma release assay (IGRA) results (IDT^−^/IGRA^+^, IDT^+^/IGRA^−^, IDT^+^/IGRA^+^). Bars represent the percentage of positive sera for each antigen: CFP10 (purple), ESAT6 (pink), MPB70 (blue), MPB83 (dark blue), P22 (light blue), and PPDB (dark purple).

In the absence of *post mortem* confirmation, IDT^+^/IGRA^−^ animals showed low serological reactivity (maximum 12%) (Figure 1A). In contrast, IDT^−^/IGRA^+^ animals exhibited a broader antibody response, with 39% testing positive for MPB70 and PPDB. The IDT^+^/IGRA^+^ group showed the highest seroreactivity, reaching 52% for PPDB, 49% for MPB70, and 46% for MPB83.

In contrast, among animals with confirmed *post mortem* lesions (Post M^+^) (Figure 1B), seroreactivity was higher overall, even in cases with discordant *intra vitam* test results. In the IDT^+^/IGRA^−^ group, 71% of samples reacted to at least one antigen, with 10 sera reactive to MPB70, 4 to MPB83, 4 to ESAT6, 3 to CFP10, 5 to PPDB, and 3 to P22. Similarly, in the IDT^−^/IGRA^+^ group, 64% of samples were reactive to MPB70, 67% to PPDB, and 69% to P22, with 23, 24, and 25 reactive samples, respectively, and lower reactivity observed to MPB83 (14 samples), ESAT6 (8 samples), and CFP10 (8 samples).

Preliminary statistical analysis comparing the two discordant groups (IDT^+^/IGRA^−^ vs. IDT^−^/IGRA^+^) in animals with PM^+^, revealed a significantly higher proportion of P22-positive animals in the IDT^−^/IGRA^+^ group (69.4%) compared to the IDT^+^/IGRA^−^ group (21.4%) (Fisher’s exact test, *p* = 0.01) (Table 6). For the remaining antigens, no significant differences were found between the two groups.

**Table 6:** ELISA results in discordant IDT/IGRA test.

Antigene	IDT^+^/IGRA^−^	% IDT^+^	IDT^−^/IGRA^+^	%IDT^−^	Fisher_p
MPB70	10	71.4	23	63.9	0.74
MPB83	4	28.6	14	38.9	0.74
ESAT6	4	28.6	8	22.2	0.72
CFP10	3	21,4	6	16.7	0.70
PPDB	8	57.1	24	66.7	0.53
P22	3	21,4	25	69.4	0.01

These findings confirm that the multi-antigen ELISA can detect humoral responses in infected animals even when traditional *intra vitam* tests fail to identify infection, and that P22, in particular, may have added value in differentiating immune profiles within discordant diagnostic categories. This highlights the potential of serology to enhance bTB surveillance and improve case detection, especially in buffaloes.

In animals with a PWM value < 0.5 (*n* = 44), serological responses were evaluated using the multi-antigen ELISA (Table 7). Within this group, particular attention was given to the 9 animals classified as IGRA invalid, for whom the low PWM rendered the IGRA result invalid. In these cases, antibody reactivity was notably high: 78% of samples were reactive to both MPB70 and PPDB, 67% to MPB83 and P22. For comparison, in the IGRA^+^ subgroup (*n* = 35), reactivity levels were lower overall. These findings suggest that the ELISA may offer valuable diagnostic insights, especially when the IGRA result is not conclusive due to an invalid response.

**Table 7:** ELISA results in samples with PWM < 0.5.

IGRA sample group	Samples	MPB70	MPB83	ESAT6	CFP10	PPDB	P22
PWM < 0,5	44	22 (50%)	13 (30%)	12 (27%)	7 (16%)	26 (59%)	27 (61%)
IGRA^+^	35	15 (43%)	7 (20%)	8 (23%)	5 (14%)	19 (54%)	21 (60%)
IGRA invalid	9	7 (78%)	6 (67%)	4 (44%)	2 (22%)	7 (78%)	6 (67%)

## 4 Discussion

Bovine TB remains a major concern in livestock farming, not only due to its economic impact linked to stamping out measures, movement restrictions, and trade limitations, but also because of the complex pathogenesis of the disease and the diagnostic challenges it poses. The traditional *intra vitam* diagnostic tools, such as the IDT and the IGRA, though widely used, can yield inconclusive or discordant results, particularly in species including the water buffalo (*B. bubalis*), where the performance of such tests is less well validated than in cattle (Martucciello et al., 2020; Kumar et al., 2024).

The diagnostic potential of a multi-antigen ELISA was evaluated by comparing its results with those from various *intra vitam* (IDT and IGRA) and *post mortem* tests. To our knowledge, this is the first study to assess the diagnostic potential of recombinant antigens such as MPB70, MPB83, ESAT6, CFP10, and protein complexes as PPDB and P22 in buffaloes for the serological detection of TB. This study is particularly important considering the current lack of commercial multi-antigen ELISA kits specifically validated for detecting *M. bovis* antibodies in buffalo species. Although van der Heijden et al. (2020) demonstrated the use of the IDEXX kit in African buffaloes (*Syncerus caffer*), their findings revealed that the reactivity of anti-bovine conjugates is significantly reduced in buffalo serum compared to cattle, which may explain the lower Se observed and further emphasizes the necessity for species-specific test validation in water buffalo. In the present study, an in-house ELISA was developed using a secondary antibody compatible with water buffalo IgG, ensuring accurate and reproducible detection of specific antibodies. Moreover, Italian legislation on bovine tuberculosis control (Gazzetta Ufficiale, 2024) recommends the use of serological tests as auxiliary tools to complement official cell-mediated immunity based methods. According to the law, they can be applied to identify anergic animals unresponsive to intradermal tuberculin or interferon-gamma assays, to aid in the diagnosis of clinically suspected cases, and to identify animals more likely to present tuberculous lesions at *post mortem* inspection. Although not considered official diagnostic tests, their role is recognized within eradication and surveillance programmes, with preference given to WOAH-validated kits. In this perspective, the development of species-adapted and multi-antigen serological assays, such as the one presented here, could represent a valuable complement to existing diagnostic tools, particularly in the framework of eradication programmes in buffalo herds.

Our study included a large and diverse sample population (*n* = 895), selected from different farms and regions, to ensure broad representation of immunological and environmental variability.

The analysis revealed that protein complexes, particularly PPDB and P22, showed the best diagnostic performance among the tested antigens. When Sp was fixed at 98.2% to maximize reliability in field conditions, PPDB retained the highest Se (63.4%), followed closely by P22 (61.3%) and MPB70 (58.7%). These results are consistent with previous observations in cattle (Infantes-Lorenzo et al., 2017; Fontana et al., 2018) and highlight the potential of PPD-derived multiprotein complexes as robust targets for serological detection of bTB.

Although MPB70 exhibited the highest Se at the optimal cut-off (Youden index), its Sp was comparatively lower. In contrast, P22, a protein complex derived from PPDB, demonstrated a better balance between Se and Sp and showed the most stable diagnostic behavior across different analyses, including a high level of agreement with the reference classification and a balanced distribution of false positives and negatives (McNemar *p* = 0.9195). These findings underline P22 as a promising candidate for the development of field-applicable serological tests in buffalo. Cohen’s Kappa statistics confirmed substantial agreement between ELISA results and reference classification for PPDB, P22, and MPB70, while CFP10 showed poor concordance, indicating lower diagnostic value. The assay demonstrated acceptable repeatability, with coefficients of variation within established thresholds, supporting the reliability of the multi-antigen ELISA.

In comparison with other *intra vitam* diagnostic tests in buffaloes, a previous study conducted in Brazil by Albernaz et al. (2015) reported a Se of 71.4% and a Sp of 82.6% for the IDT in this species. Similarly, Martucciello et al. (2020) evaluated the IGRA in buffaloes, reporting Se values ranging from 75.3% to 98.4% and Sp values from 94.3% to 98.5%. While these Se values appear higher than those obtained in our serological assay, both IDT and IGRA present several technical limitations. The IDT, which has been instrumental in the control and eradication programmes of bTB in cattle in several countries, results in a subjective test with poor Se and Sp in buffalo (van der Heijden et al., 2020), especially in very early stages of infections, rather than in extremely chronic cases (Albernaz et al., 2015, Kanameda et al., 1999). The IGRA test has good accuracy, is an objective test with a short run time and can be repeated without time limitations, as no tuberculin is inoculated (de la Rua-Domenech et al., 2006). However, the IGRA test presents logistical difficulties as it requires blood samples to be transported under controlled temperature conditions (approximately 10–26 °C) and incubated with antigens within a few hours of collection (Rothel et al., 1992). Delays in stimulating the samples can drastically reduce the test’s Se (Gormley et al., 2004). In this context, serology, by assessing the humoral immune response, may provide complementary diagnostic information, particularly in cases of inconclusive or discordant IDT and IGRA results. In addition, this assay offers the advantage of allowing samples to be stored for several days before testing, which greatly facilitates logistical planning both in the herds and in the laboratory. This flexibility enables the collection of numerous samples in a single day and allows testing to be performed even the day after sampling, thanks to a method that is simple, rapid, and cost-effective. Furthermore, in areas where serological testing for other diseases, such as brucellosis, is already performed, the same sample could also be used to screen for bTB using the ELISA test. In the case of a positive result, follow-up testing with in-herd diagnostics such as the IDT and IGRA could then be considered. The use of ELISA could greatly facilitate the work of veterinary services, especially in regions where logistical challenges make it difficult to gather and restrain animals for IDT or where access to laboratories capable of performing the IGRA is limited. This approach would allow for additional screening opportunities that complement tests based on CMI.

In animals with positive *intra vitam* tests but lacking post-mortem confirmation (IV+/ PM−), a relevant proportion showed seroreactivity, especially to PPDB (43%) and MPB70 (41%). These findings suggest that serology may detect infected animals missed by traditional tests, especially in early or subclinical stages, or in cases of sampling/processing errors that may limit the Se of post-mortem diagnostics.

In animals with confirmed infection (IV+/ PM+), the reactivity was significantly higher across all antigens, particularly for PPDB, MPB70, ESAT6, and P22 (Fisher’s exact test *p* < 0.01). McNemar’s test confirmed directional discordance (*p* < 0.001), indicating that the ELISA may capture aspects of the immune response not detected by IDT or IGRA. Among the tested antigens, P22 consistently showed the best overall performance, both alone and in combination with other antigens, supporting its role as a valuable component in multi-antigen ELISA formats. Despite low Kappa values, which indicate poor agreement between ELISA and cell-mediated immunity-based tests beyond chance, these results suggest that the ELISA may detect antibodies not identified as positive by IDT and IGRA. This highlights the complementary role of antibody detection in bTB surveillance.

These findings are consistent with those reported by Waters et al. (2011) in cattle, where serological responses were notably stronger in animals with confirmed infection, highlighting the potential of serology for identifying more advanced stages of disease. Similarly, Fontana et al. (2018) observed in cattle that a significant proportion of animals with positive *post mortem* findings, but negative *intra vitam* results, exhibited detectable antibody levels, suggesting that humoral responses may persist even during early or latent stages of infection. In fact, the humoral immune response against *M. bovis* is very complex and antibody titres can be subject to important variations in the course of infection. They are mainly produced in the advanced stages of bTB infection (Pollock and Neill, 2002), but an early antibody response has also been reported in experimentally infected cattle (Waters et al., 2006).

Serological profiles varied in animals with discordant IDT and IGRA results. Among buffaloes with IDT^+^/IGRA^−^ results and no post-mortem confirmation, reactivity was low, with only 12% of samples reactive to MPB70, MPB83, and PPDB. Conversely, in IGRA^+^/ IDT^−^ animals, broader serological reactivity was observed, particularly against MPB70 (39%), MPB83 (35%), and PPDB (39%). This pattern mirrors results found in cattle by Waters et al. (2011) who observed higher reactivity in IGRA^+^/IDT^−^ cattle. These findings suggest that differences in immune responses between cell-mediated immunity (detected by IGRA) and humoral immunity (captured by serology) may contribute to discordant results. The ability of the ELISA to detect humoral responses in IGRA^+^/IDT^−^ animals highlights the potential for serological testing to complement cell-mediated tests in diagnosing bTB, particularly in cases where traditional diagnostic methods provide inconclusive results.

Notably, among animals with *post mortem* confirmation (PM^+^) but discordant *intra vitam* results, seroreactivity was substantially higher. In the IDT^+^/IGRA^−^ group, 71% of buffaloes showed reactivity to MPB70 and 36% to at least one antigen, and 64%–69% of IGRA^+^/IDT^−^ buffaloes responded to MPB70, PPDB, or P22.

Interestingly, P22 demonstrated potential in differentiating immune profiles between discordant categories: in PM^+^ animals, seroreactivity to P22 was significantly higher in the IGRA^+^/ IDT^−^ group (69.4%) compared to the IDT^+^/IGRA^−^ group (21.4%) (*p* = 0.01). This may indicate that P22 could help to stratify infection stages or immune responses not captured by IGRA or IDT alone.

The multi-antigen ELISA also demonstrated diagnostic value in animals with invalid IGRA results (PWM < 0.5; *n* = 9). Despite an indeterminate status, serological responses were detected in a substantial proportion of buffaloes, particularly against PPDB and MPB70 (78%) and P22 and MPB83 (67%).

This result suggests that ELISA can serve as a valuable diagnostic tool when IGRA cannot be interpreted, either due to technical issues or poor sample quality.

Taken together, these findings highlight the utility of the multi-antigen ELISA as a complementary diagnostic tool for TB in water buffaloes, capable of identifying infected animals across a range of clinical and immunological presentations. Its ability to detect serological responses in animals with uncertain or discordant *intra vitam* and *post mortem* results enhances its value in surveillance and eradication programmes, particularly in regions where traditional diagnostic methods may fail to provide definitive outcomes. Although the ELISA cannot be used as a stand-alone diagnostic test, combining serological testing with cell-mediated immunity-based methods may increase the detection of infection and enhance control and eradication programmes. Beyond water buffalo, species-adapted multi-antigen ELISAs could also be applied to wildlife and other reared species (EURL Bovine Tuberculosis), contributing to a One Health approach that addresses the human–animal–environment interface. Overall, reliable and cost-effective serological assays offer valuable opportunities to complement official diagnostics and strengthen TB surveillance across species.

## Data Availability

The original contributions presented in the study are included in the article/Supplementary material, further inquiries can be directed to the corresponding author/s.
