# Supplementary material for: Evaluation of serological assays for intra vitam diagnosis of bovine tuberculosis in water buffalo (Bubalus bubalis)
Source: Front Microbiol. 2025 Nov 12;16:1684425. doi: 10.3389/fmicb.2025.1684425 (PMC12646987; doi:10.3389/fmicb.2025.1684425)
Supplement: Supplementary file 1 [file Data_Sheet_1.docx]

Supplementary Material

# Supplementary Tables

Table S1. Sensitivity (Se) and Specificity (Sp) of combinations of antigens

|  | TB POSITIVE | | TB FREE | |  | |  |  |
| --- | --- | --- | --- | --- | --- | --- | --- | --- |
|  | POSITIVE | NEGATIVE | POSITIVE | NEGATIVE | SE (CI 95%) | SP (CI 95%) | | ACCURACY |
| MPB70 | 202 | 142 | 8 | 385 | 0,59 (0,64±0,54) | 0,98 (0,99±0,97) | | 0,8 |
| MPB83 | 143 | 201 | 7 | 386 | 0,42 (0,47±0,37) | 0,98 (0,99±0,97) | | 0,72 |
| ESAT6 | 113 | 231 | 7 | 386 | 0,33 (0,38±0,28) | 0,98 (0,99±0,97) | | 0,68 |
| CFP10 | 48 | 296 | 7 | 386 | 0,14 (0,18±0,10) | 0,98 (0,99±0,97) | | 0,59 |
| PPDB | 218 | 126 | 7 | 386 | 0,63 (0,68±0,58) | 0,98 (0,99±0,97) | | 0,82 |
| P22 | 211 | 133 | 8 | 385 | 0,61(0,66±0,56) | 0,98 (0,99±0,97) | | 0,81 |
| MPB70/MPB83 | 206 | 138 | 14 | 379 | 0,6 (0,65±0,55) | 0,96 (0,98±0,94) | | 0,79 |
| MPB70/ESAT6 | 213 | 131 | 15 | 378 | 0,62 (0,67±0,57) | 0,96 (0,98±0,94) | | 0,8 |
| MPB70/CFP10 | 206 | 138 | 14 | 379 | 0,6 (0,65±0,55) | 0,96 (0,98±0,94) | | 0,79 |
| MPB70/PPDB | 229 | 115 | 12 | 381 | 0,67 (0,72±0,62) | 0,97 (0,99±0,95) | | 0,83 |
| MPB70/P22 | 233 | 111 | 15 | 378 | 0,68 (0,73±0,63) | 0,96 (0,98±0,94) | | 0,83 |
| MPB83/ESAT6 | 167 | 177 | 13 | 380 | 0,49 (0,54±0,44) | 0,97 (0,99±0,95) | | 0,74 |
| MPB83/CFP10 | 154 | 190 | 14 | 379 | 0,45 (0,50±0,40) | 0,96 (0,98±0,94) | | 0,72 |
| MPB83/PPDB | 224 | 120 | 14 | 379 | 0,65 (0,70±0,60) | 0,96 (0,98±0,94) | | 0,82 |
| MPB83/P22 | 221 | 123 | 14 | 379 | 0,64 (0,69±0,59) | 0,96 (0,98±0,94) | | 0,81 |
| ESAT6/CFP10 | 121 | 223 | 14 | 379 | 0,35 (0,40±0,30) | 0,96 (0,98±0,94) | | 0,68 |
| ESAT6/PPDB | 224 | 120 | 14 | 379 | 0,65 (0,70±0,60) | 0,96 (0,98±0,94) | | 0,82 |
| ESAT6/P22 | 223 | 121 | 15 | 378 | 0,65 (0,70±0,60) | 0,96 (0,98±0,94) | | 0,82 |
| CFP10/PPDB | 219 | 125 | 14 | 379 | 0,64 (0,69±0,59) | 0,96 (0,98±0,94) | | 0,81 |
| CFP10/P22 | 215 | 129 | 15 | 378 | 0,63 (0,68±0,58) | 0,96 (0,98±0,94) | | 0,8 |
| PPDB/P22 | 238 | 106 | 12 | 381 | 0,69 (0,74±0,64) | 0,97 (0,99±0,95) | | 0,84 |
| MPB70/MPB83/ESAT6/CPF10 | 220 | 124 | 26 | 367 | 0,64 (0,69±0,59) | 0,93(0,96±0,90) | | 0,8 |
| ESAT6/CFP10/P22 | 227 | 117 | 22 | 371 | 0,66 (0,71±0,61) | 0,94 (0,96±0,92) | | 0,81 |

This table shows single antigens and their combinations, with the corresponding Se and Sp data, including the 95% confidence interval, and accuracy.

**Table S2. Agreement between the ELISA test results and the reference classification (true positives and true negatives), assessed using Cohen’s Kappa coefficient and McNemar’s test for each of the six antigens.**

| Antigen | Kappa | Lower CI95% | Upper CI 95% | McNemar pvalue |
| --- | --- | --- | --- | --- |
| MPB70 | 0.719 | 0.669 | 0.769 | 0 |
| MPB83 | 0.565 | 0.505 | 0.624 | 0.3845 |
| ESAT6 | 0.484 | 0.421 | 0.546 | 0 |
| CFP10 | 0.22 | 0.156 | 0.284 | 0 |
| PPDB | 0.796 | 0.753 | 0.84 | 0.0111 |
| P22 | 0.733 | 0.684 | 0.782 | 0.9195 |

Kappa values indicate the strength of agreement beyond chance, with 95% confidence intervals reported. McNemar’s test was used to evaluate asymmetry in misclassification errors (false positives vs. false negatives); a non-significant p-value (p > 0.05) indicates a balanced distribution of discordant pairs.

**Table S3.** **Reactivity to single or multiple antigens**

| **Reactivity to single or multiple antigens** | |
| --- | --- |
| No. antigens | No. samples |
| 1 | 253 |
| 2 | 217 |
| 3 | 192 |
| 4 | 145 |
| 5 | 94 |
| 6 | 34 |

**Table S4.**  **Frequency of positive responses to pairs of antigens among TB-positive sera**

|  | TB POSITIVE | % (TOT. 344) |
| --- | --- | --- |
| MPB70/MPB83 | 139 | 40.41% |
| MPB70/ESAT6 | 102 | 29.65% |
| MPB70/CFP10 | 44 | 12.79% |
| MPB70/PPDB | 191 | 55.52% |
| MPB70/P22 | 180 | 52.33% |
| MPB83/ESAT6 | 89 | 25.87% |
| MPB83/CFP10 | 37 | 10.76% |
| MPB83/PPDB | 137 | 39.83% |
| MPB83/P22 | 133 | 38.66% |
| ESAT6/CFP10 | 40 | 11.63% |
| ESAT6/PPDB | 107 | 31.10% |
| ESAT6/P22 | 101 | 29.36% |
| CFP10/PPDB | 47 | 13.66% |
| CFP10/P22 | 45 | 13.08% |
| PPDB/P22 | 191 | 55.52% |
| MPB70/MPB83/ESAT6/CPF10 | 34 | 9.88% |
| ESAT6/CFP10/P22 | 40 | 11.63% |
